# Characteristics of paroxysmal nocturnal hemoglobinuria patients in Brazil: A retrospective administrative claims database analysis of PNH patients in Brazilian public healthcare system

**DOI:** 10.1371/journal.pone.0288708

**Published:** 2023-07-26

**Authors:** Sandra Fatima Menosi Gualandro, Marco Aurélio Salvino, Lucas Bassolli de Oliveira Alves, Thainá Jehá

**Affiliations:** 1 University of São Paulo Medical School, São Paulo, São Paulo, Brazil; 2 PPGMS-Federal University of Bahia, Salvador, Bahia, Brazil; 3 University of São Paulo, São Paulo, São Paulo, Brazil; 4 Alexion Pharmaceuticals, São Paulo, São Paulo, Brazil; University of Buea, CAMEROON

## Abstract

**Introduction:**

Few studies have reported the profile of patients with paroxysmal nocturnal hemoglobinuria (PNH) and their care in the Brazilian health system.

**Objective:**

To describe clinical and epidemiological characteristics of patients with PNH in the Brazilian public health system including procedures performed, associated comorbidities and visits to health care professionals.

**Methods:**

In a real-world observational, retrospective, population-based cohort study, anonymized secondary data provided by the Department of Informatics of the Brazilian Unified Health System (DATASUS) were analyzed. Patients were considered eligible if they had at least one procedure coded with the ICD-10 code D59.5 from January 1, 2008 to December 30, 2018.

**Results:**

In total, 675 individual PNH patients were identified (52.4% female; prevalence of 1:237,000 people). Around 15.8% of the patients included had myelodysplastic syndrome and about half of the sample had other aplastic anemias and/or other bone marrow failure syndromes. Portal vein thrombosis (I82 ICD code) was reported in 4.3% of patients. Regarding hospitalizations, 263 individual PNH patients had 416 inpatient admissions with the ICD code for PNH (D59.5) on admission. Twelve deaths occurred during the study period, of which two had the PNH ICD code related with the cause of death, while another three deaths were associated with acquired hemolytic anemia (D59.9), unspecified aplastic anemia (D61.9) and acute respiratory failure (J96.0), respectively.

**Conclusion:**

Despite its limitations, this statistical analysis of data extracted from DATASUS reasonably describes PNH patients in Brazil and its variations across different regions of the country. Comorbidities frequently associated with PNH such as portal vein thrombosis were not as common in our study, but it is assumed that several thrombotic events at specific sites were coded under the broader I82 ICD code. The frequency of visits to different health professionals, including hematologists, increased after the diagnosis of PNH. Among hospitalized PNH patients, the mortality rate was 4.5%.

## Introduction

Paroxysmal Nocturnal Hemoglobinuria (PNH) is a rare acquired clonal hematopoietic stem cell disorder characterized by intravascular hemolysis, hemoglobinuria, anemia, and thrombosis [1]. PNH annual incidence is estimated to be 1–9 cases per 100,000 people worldwide, but it might be higher in certain regions [2, 3]. PNH is rare in children, most cases emerge during adolescence and the highest prevalence occurs among people aged 30–59 years, with 54.6% of cases reported in this age range [4–8].

In the past, PNH was considered a benign hematological disorder, its association with significant morbidity and mortality has long been recognized. Retrospective evidence suggests a mortality rate of 35% within 5 years from diagnosis, reaching 50% after 10 years regardless the best supportive care provided [9]. Thrombosis is the most serious complication and the leading cause of death among PNH patients, accounting for 40–67% of deaths. Potential complications such as deep vein thrombosis or pulmonary thromboembolism can result in significant morbidity or even sudden death [10]. PNH is also associated to Budd-Chiari syndrome, myocardial infarction, and stroke [3, 11, 12]. Other disabling morbidities that PNH patients may develop include renal failure, pulmonary hypertension, erectile dysfunction, and dysphagia [4, 9, 10, 12–15].

When PNH is suspected based on clinical and laboratory data, the diagnosis is relatively straightforward, as deficiency of glycosylphosphatidylinositol-anchored proteins (GPI-Aps) on peripheral blood cells can be readily demonstrated by flow cytometry. PNH is clinically heterogeneous and patients may present with both subjective and objective signs and symptoms such as fatigue, lethargy, asthenia, dyspnea, abdominal pain, chest pain, erectile dysfunction, odynophagia, headache, hemoglobinuria, jaundice, and thromboembolic events. In addition, some abnormal laboratory results may be present, such as thrombocytopenia, elevated levels of lactate dehydrogenase (LDH), low haptoglobin, high indirect bilirubin, iron deficiency, and hemosiderinuria [16].

Although much progress has been made in understanding the pathophysiology and management of the disease in recent years, comprehensive research is needed to better describe both the epidemiological profile of PNH patients in the Brazilian public health care system. For example, the typical burden of comorbidities, outpatient procedures and hospitalizations among patients with PNH are not well known, as is the frequency of their visits to health care professionals before and after being diagnosed. In the Brazilian context, such data can be obtained from the DATASUS. DATASUS is a large public database that provides data on the use of health resources and services by approximately 160 million people, corresponding to about 75% of the Brazilian population, who rely exclusively or mainly on the public system for access to health care. DATASUS is based on administrative claims from both outpatient and in-hospital procedures within the scope of the public health system in Brazil.

As PNH is a rare condition, retrospective studies using large databases can provide a unique perspective on patient profiles and what tests and procedures were performed for diagnostic purposes, as well as the type of treatment prescribed. Therefore, this study aims to describe the main clinical and epidemiological characteristics in this population (age, sex, geographic region, and associated comorbidities), as well as their health care (including procedures and visits to health professionals), and estimate the hemolysis-related in-hospital mortality among PNH patients.

## Materials and methods

### Design and source of data

This is a real-world observational, retrospective, population-based cohort study, based on the analysis of anonymized secondary data provided by the DATASUS, originating from two separate data systems: Hospital Admissions Information System (SIH) and Outpatient Procedures Information Systems (SIA) [17, 18]. Both SIH and SIA are nationwide administrative claim databases that aggregate data on hospital admissions and outpatient procedures performed in the Brazilian Unified Public Health Care System (SUS). The SIH database contains data on demographics, medical diagnosis, main reason for hospitalization, length of stay, procedures performed during the admission period, and in-hospital mortality. SIA data refer to outpatient care (medical visits, laboratory tests, outpatient medical procedures, treatments, etc.), including demographics, diagnosis, and specialty of attending physicians [17, 18]. Both databases are frequently used in Brazil as a subsidy for planning health policies and also for research purposes [19].

Data from SIA and SIH were not cross-referenced because it is not possible to identify a patient’s record number in the SIH. This means that: 1) All patients in the sample of this study come from the SIA; and 2) It is possible to identify the number of patients who had at least one hospital admission event, but it is not possible to find out who these patients were individually.

For this research, analytical and medical writing support was provided by Techtrials, a Brazilian company that has been providing services in the area of health data since its foundation in 2009. With experience gained in observational study projects and using real-world health care data, Techtrials developed a proprietary structured health data platform, called TT RWD. Data extracted from the SIA and SIH were pre-structured on the TT RWD platform, which automatically collects anonymized and publicly available data by using electronic robots (called “ETLs” and “web crawlers”). Therefore, for this study, data extraction and application of filters relevant to our research objectives were performed by Techtrials.

### Data management

Techtrials was responsible for all data management. It was carried out according to Techtrials protocols, which were written, submitted to and approved by Alexion Pharmaceuticals prior to the start of the analysis. All databases released by DATASUS are anonymized using an encrypted patient code. All Techtrials databases are physically stored on the Microsoft Azure Cloud, and appropriate actions have been taken for data backup and system stability. The TT RWD Platform ensures patient confidentiality, as well as data security and confidentiality throughout the study. The TT RWD Platform is only accessible via secure login and password access with a two-step authentication (please visit https://www.powerbi.com/home).

### Study population and sample

The global study population is composed of PNH cases reported in Brazil. Patients were considered eligible for inclusion if they had at least one procedure coded with the World Health Organization ICD-10 [20] code D59.5 in either the SIH or SIA from January 1, 2008 to December 30, 2018 (this period refers to the day when data were entered in DATASUS). PNH cases reported outside this period were excluded. All eligible records were abstracted and analyzed; therefore, a sample size calculation was not performed. As the DATASUS database covers all Brazilian SUS records, the final sample can be considered representative of the target population across the country.

### Data extraction

Files containing anonymized data from all 26 States and the Federal District of Brazil were extracted directly from the DATASUS website, by using the ICD code D59.5 for PNH. Data were then disaggregated by state, sex, age group, and diagnosis codes. An official DATASUS data dictionary was used to understand the intrinsic characteristics of the database and identification of selected fields that could be relevant to the objectives of the study. These are unique data assets, as they have little attrition, include large, continuously enrolled populations, and record the use of health care resources and costs from a single and universal payer [17, 18].

### Analysis

The following variables were included in the analysis of patients who had at least one procedure recorded with the ICD code D59.5: age, sex, geographic region, health care professionals (HCP) visited, and presence of comorbidities (the most frequent comorbidities in patients with PNH, as well as other clinical conditions less related to the disease). Selected comorbidities were addressed using previously defined ICD codes for complications and conditions commonly associated with PNH (D46, myelodysplastic syndrome; D61, other aplastic anemias and other bone marrow failure syndromes; I81, portal vein thrombosis; I82, other venous embolism and thrombosis). This predefined list of ICD codes was used to track back both inpatient and outpatient care records during the history of each patient in the database. If a patient had at least one procedure coded with any of these ICD codes, it was used to assume a comorbid diagnosis. The time of PNH diagnosis was defined as the first date on which a given patient had a procedure entered in the SIA database with the ICD code D59.5 (index date). A before-and-after analysis was performed using this index date as a reference to explore whether the medical specialties recorded for the patients were different after the PNH diagnosis. All relevant demographic characteristics available in the database were descriptively analyzed. For categorical data, measures of frequency, contingency tables and charts were used for data visualization and report. Continuous variables were described using measures of central tendency and dispersion. Statistical analysis was performed using RStudio version 3.5.2.

### Ethics

This study does not involve the participation of human subjects. This is a research based on the analysis of anonymized, publicly available secondary data from DATASUS. This large database concentrates public health care data in Brazil and can be accessed at https://datasus.saude.gov.br/ Therefore, approval by an institutional review board (ethics committee) and written informed consent provided by patients are not required.

## Results

After data processing, 675 individual patients with PNH were identified (52.4% female, mean age 44.2 years; prevalence of 1:237,000 people). Almost 30% of patients were between 21 and 35 years old in 2021 and about 60% of cases were from the Southeast Region. Patient characteristics are summarized in Table 1.

**Table 1:** Characteristics of PNH patients.

Variable	*n* (%)
**Female**	355 (52.5)
**Age (mean ± SD)**	44.2 ± 18.6
**Age group** ^a^	
0 to 10 years	21 (3.1)
11 to 20 years	60 (8.8)
21 to 35 years	198 (29.3)
36 to 45 years	125 (18.5)
46 to 60 years	134 (19.8)
61 to 80 years	119 (17.6)
81+ years	18 (2.6)
**Geographic region**	
North	23 (3.4)
Northeast	106 (15.7)
Midwest	31 (4.5)
Southeast	399 (59.1)
South	116 (17.1)
**Skin color or race/ethnicity**	
White	427 (63.2)
Mixed race	89 (13.2)
Black	34 (5)
Asian	16 (2.3)
No information	109 (16.1)

^a^ Age groups in 2021.

**Fig 1:**
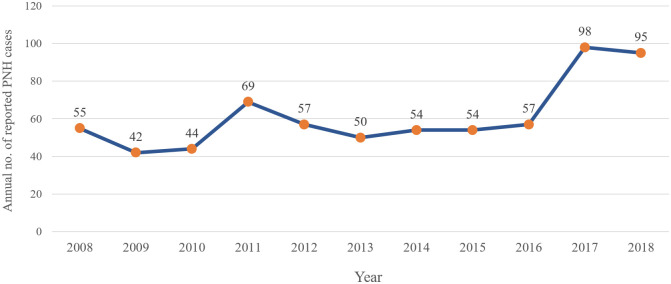
PNH cases reported for each year of the study period.

Fig 1 illustrates the occurrence of PNH cases during the study period. This data corresponds to the first time a PNH record was identified in DATASUS for a given patient. About 30% of cases were reported in the 2017–2018 period.

**Fig 2:**
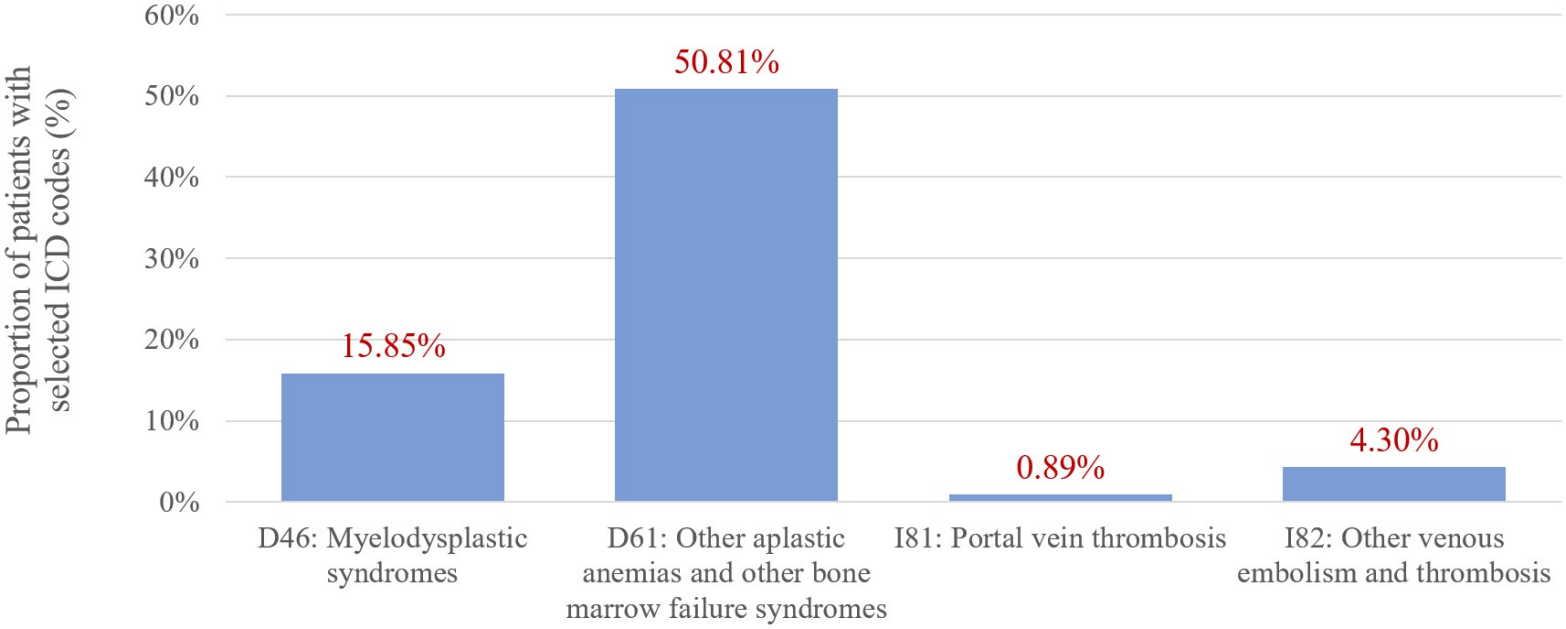
Frequency of selected PNH-related comorbidities, grouped.

**Fig 3:**
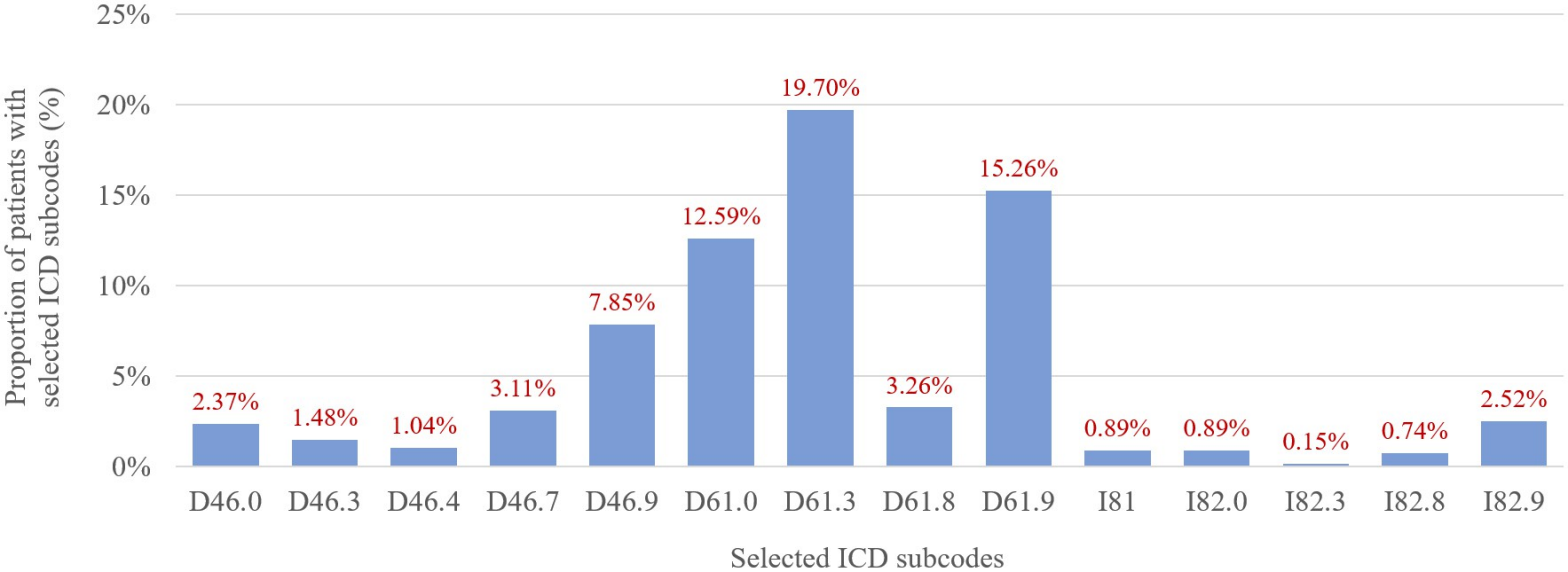
Frequency of selected PNH-related comorbidities, separated by ICD subcodes. **D46.0,** Refractory anaemia without sideroblasts; **D46.3,** Refractory anaemia with excess of blasts with transformation; **D46.4,** Refractory anaemia, unspecified; **D46.7,** Other myelodysplastic syndromes; **D46.9,** Myelodysplastic syndrome, unspecified; **D61.0,** Constitutional aplastic anaemia; **D61.3,** Idiopathic aplastic anaemia; **D61.8,** Other specified aplastic anaemias; **D61.9,** Aplastic anaemia, unspecified; **I81,** Portal vein thrombosis; **I82.0,** Budd-Chiari syndrome; **I82.3,** Embolism and thrombosis of renal vein; **I82.8,** Embolism and thrombosis of other specified veins; **I82.9,** Embolism and thrombosis of unspecified vein.

Figs 2 and 3 illustrate the proportion of patients with at least one event coded as a selected PNH-related comorbidity during the study period. About 15.8% of patients had myelodysplastic syndrome and about half of the sample had other aplastic anemias and/or other bone marrow failure syndromes. Portal vein thrombosis (I81 ICD code) was reported in 0.89%, less than expected in PNH patients, but it is possible that this comorbidity was coded as I82 (other venous embolism and thrombosis), which had a frequency of 4.3% in this population.

**Fig 4:**
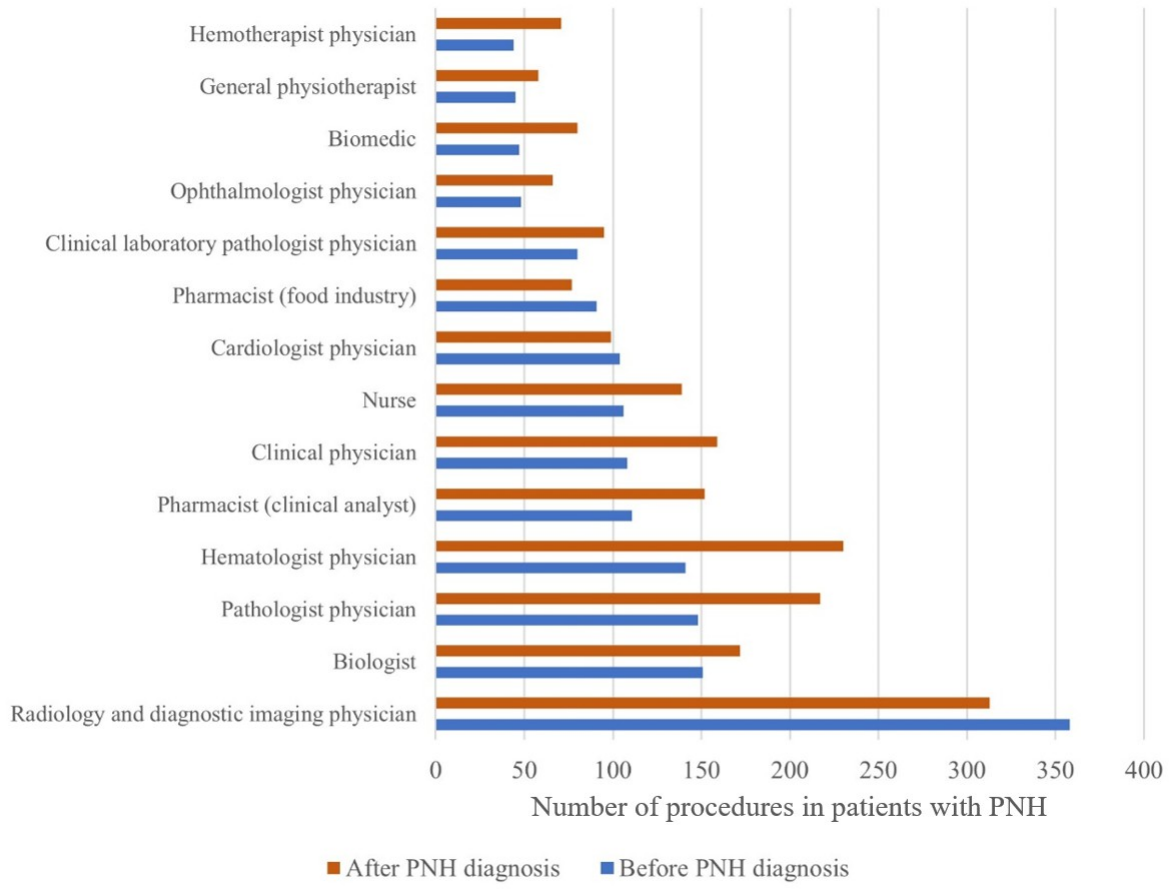
Before-and-after analysis of health professionals responsible for visits of procedures in patients with PNH from the database.

Fig 4 presents the different health care providers visited before and after the diagnosis of PNH. In this analysis, 545 PNH patients who had events before and after the index data were included. Visits of examinations by radiologists, hematologists, and pathologists were the most common both before and after PNH diagnosis, and almost all specialty categories had a numerical increase after diagnosis, particularly hematologists and pathologists.

Chronic viral hepatitis C, followed by idiopathic aplastic anemia, and unspecified aplastic anemia were the most frequent comorbidities in the study population, both before and after PNH diagnosis.

As for hospital admissions, 263 patients with PNH had 416 hospitalizations with a code corresponding to the disease (D59.5). In the Hospital Admissions Information System (SIH), from which hospital admission and death data were extracted, there are no patient identification numbers, which would allow them to be correlated with patients in the SIA system. Therefore, it is not possible to state that all 263 hospitalized during the study period were part of the 675 outpatients counted in the study sample. In terms of in-hospital mortality, twelve deaths occurred during the study period, of which two had the PNH ICD code related with the cause of death and another three deaths were associated with acquired hemolytic anemia (D59.9), unspecified aplastic anemia (D61.9) and acute respiratory failure (J96.0), respectively. Seven deaths were not associated with any PNH-related ICD code.

## Discussion

We estimate a current prevalence of PNH at 1:237,000 in the population covered by the Brazilian public health system. This estimate may be relevant, given the lack of data on the prevalence of this disease in South America. A few studies have previously reported PNH case series in Brazil, mostly in single-center reports. In 2011, by Brito Jr. et al. reported nine patients identified with PNH by flow cytometry in a sample of 30 suspected cases in the city of Belém, Pará [21]. At the 2019 American Society of Hematology annual meeting, Yamakawa et al. presented data from 109 patients with a PNH clone treated at two centers in the city of São Paulo, SP, of which 56 had hemolytic disease [22]. In 2021, de Azambuja et al. reported a retrospective cohort of 167 PNH patients and the impact of disease burden on long-term follow-up [23]. Pires da Silva et al. published a retrospective series of 87 patients with PNH detected by flow cytometry between 2006 and 2019 at a single Brazilian referral center, highlighting the highly variable clinical presentation of the disease [24].

We have found 675 PNH individual patients according to the criteria defined for the present analysis, based on ICD codes recorded in the SIA outpatient database. In this sample, the distribution by sex and age was similar to that described for patients enrolled in the International PNH Registry (median age 44 years, 52.4% female) [25]. Most patients in our sample were treated in the Southeast region, the most populous and the one with the highest concentration of physicians in the country [26]. These regional characteristics may explain the higher concentration of PNH patients observed in this part of the country, as access to medical care can facilitate diagnosis and referral to specialized treatment. Patients in this sample were predominantly white (63.2%), in a proportion that exceeds that of whites in the country (42.3%) [27]. Previous publications have raised questions about potential differences in the course of PNH by ethnic groups [2].

Four comorbidities or conditions commonly associated with PNH were pre-selected for this analysis: myelodysplastic syndrome; aplastic anemias; portal vein thrombosis; and other venous embolism and thrombosis. The frequency of thromboembolic events (ICD code I82) in our sample was around 4.3%, lower than the reported rate in the International PNH Registry (16%) among 1610 patients [25]. Portal vein thrombosis was reported in less than 1% of sample during the study period, but it is reasonable to assume that events of specific thrombosis sites were coded under the broader ICD code I82.

Bone marrow disorders (BMD) are frequently reported among PNH patients. In a cohort of Asian patients, one or more BMDs was observed in 70% of all cases [28], while data from the International PNH Registry indicate a lower prevalence (43.5%) [25]. Thus, the combined prevalence of BM disorders assessed in our database analysis falls within the range of previous reports. Myelodysplastic syndromes in both mentioned cohorts was reported in 5.8–10.9% of PNH patients [25, 28], while in our data 15.8% of PNH patients in the DATASUS database had at least one admission or outpatient procedure with an ICD code for myelodysplastic syndrome. In the Asian study [29], 37.5% of PNH patients had a history of aplastic anemia, similar to our findings. Given the retrospective nature of this analysis of administrative claims data, it is not possible to rule out misclassification as an explanation for these apparent discrepancies.

As expected, we observed an increase in the proportion of patients who sought a hematologist as their primary health care provider following the diagnosis of PNH (based on the date the ICD code D595 was first entered in the database), compared to medical visits before the index date. Visits to other health care providers usually associated with laboratory and imaging tests (such as pathologists, pharmacists, biologists in laboratory tests, and radiologists in medical imaging centers) were also more common after PNH diagnosis, potentially indicating an increase in the use of such health resources.

Data on rates and reasons for hospitalization in patients with PNH are scarce in the literature. Schrezenmeier et al. reported hospitalization rates for PNH-related complications in the 6 months prior to the study enrollment. Of 856 patients, 22.7% had been hospitalized, with a higher risk of inpatient admission among those with a history of thrombosis or self-reported PNH-related symptoms (conjunctival icterus, chest pain, dysphagia, abdominal pain, hemoglobinuria, dyspnea, or fatigue) in the past six months [25]. In our cohort, 263 individual PNH patients had 416 admissions with an initial ICD code corresponding to the disease (D59.5). These patients are assumed to be a subgroup of the total records in the sample. However, as identification numbers for patients are not available in the SIH, it is not possible to state that all these patients are from the same group. The mortality rate among hospitalized patients with PNH was 4.5%.

Some limitations of our study must be discussed. The retrospective approach and the administrative nature of the DATASUS database imply a potential risk for misclassification and information bias, as ICD codes may not always be correctly entered into the primary DATASUS database. Other studies in similar populations also described this type of limitation [19]. Some clinical variables that would be helpful to better describe the characteristics and journey of patients (e.g., date of diagnosis) are not available in the inpatient and outpatient databases, which can cause uncertainty to the data. The tracking of the PNH code, as well as the codes associated with this condition, was performed using an algorithm, which makes the process safe in terms of recovering all D59.5 codes present in the DATASUS database. As it is a secondary database, however, it is not possible to know who registered the codes or to be sure of their diagnosis. This important drawback can generate some inaccuracy in the sample estimates. Despite these limitations, to our knowledge this is the first nationwide study addressing characteristics of PNH patients in Brazil, covering both demographics, comorbidities, hospitalization, and mortality. Further research combining other data sources such as medical charts, laboratory tests and patient interviews could elucidate other aspects of the disease burden and clinical course of PNH in Brazil.

## Conclusion

This may be the first epidemiological study in order to assess characteristics of PNH patients in Brazil based on data from the public health system. Despite its limitations, this statistical analysis of data extracted from DATASUS reasonably describes the epidemiological profile of these patients in Brazil, as well as its variations across different regions of the country. Comorbidities frequently associated with PNH such as portal vein thrombosis were not as common in our study, but it is assumed that several thrombotic events at specific sites may have been coded under the broader I82 ICD code. Other comorbidities increased significantly after diagnosis. The frequency of patient visits to different health care professionals, particularly hematologists, increased after being diagnosed with PNH. Among hospitalized PNH patients, the mortality rate was 4.5%.
